# Supplementary material for: Are carbon emissions trading and green financial instruments synergistic? -Comprehensive quantitative research based on content analysis
Source: PLoS One. 2024 Mar 7;19(3):e0298601. doi: 10.1371/journal.pone.0298601 (PMC10919601; doi:10.1371/journal.pone.0298601)
Supplement: S1 Data — (ZIP) [file pone.0298601.s001.zip › Data/地级市Z_F工作报告.zip!/╔┬╬≈/║║╓╨╩╨/└┤╫╘╣1⁄2╓┌║┼í╛┬φ┐╦╩2╛▌═°í┐_2016.docx]

各位代表：

　　现在，我代表市人民政府向大会作工作报告，请予审议，并请市政协委员和其他列席人员提出意见。

　　一、2015年工作和“十二五”发展回顾

　　2015年，在省委、省政府和市委的坚强领导下，全市上下以习近平总书记来陕视察为鼓舞，紧紧围绕“五个扎实”新要求和中、省部署安排，积极应对复杂宏观环境，统筹推进稳增长、调结构、促改革、惠民生、防风险各项工作，全面完成了市四届人大六次会议确定的各项目标任务。全年生产总值1064.8亿元，增长9.6%、位居全省第五；财政总收入100.2亿元，同口径增长10%；地方财政收入44.66亿元，同口径增长13.7%、位居全省第四；财政支出258.6亿元，增长10.5%、位居全省第五；全体居民人均可支配收入14378元，增长10.04%、位居全省第三。

　　（一）投资拉动成效明显。抢抓机遇争取中省投资，创造条件加快项目建设，不断完善领导包抓和现场观摩工作机制，强力推进“千亿投资工程”。全社会固定资产投资完成1039.4亿元，增长23%、位居全省第三。335个重点项目完成投资437.2亿元，占年计划的118.1%。中航集团与省政府正式签订共建汉中航空智慧新城协议，镇巴油气勘探“春生1号”井顺利下钻，金汉客车生产线、大唐略电热电扩建等项目建成投运，阳安二线、酒泉至湖南特高压线路汉中段工程等项目开工建设。组织开展京汉务实合作经贸交流和张骞文化经贸使团大篷车队宣传推介活动，积极赴粤港澳、京津冀和“中亚三国”等地招商引资，全年引资462.6亿元、增长10.9%。

　　（二）工业经济企稳回升。面对市场低靡严峻挑战，尤其是钢铁、有色、建材、化工等产业大幅下滑的不利形势，启动新一轮“工业翻番工程”，强化“平茬齐过、盘点过堂，一企一策、精准发力，多点支撑、多元带动”工作对策，引导企业精益管理、挖潜节支、稳产促销。全年实现规模以上工业总产值1053.1亿元，增长4.4%；增加值341.4亿元，增长9%、超出全省2个百分点。75个工业重点技改项目完成投资55.6亿元。大力实施“大众创业、万众创新”和“小巨人”成长计划，建成电商孵化园4个，培育电商企业60户、小微企业3167户。全年中小企业实现营业收入1640亿元、增长17%。

　　（三）农村经济健康发展。把转方式、调结构贯穿于“农业倍增工程”各个环节，依托优势壮大主导产业，搭建平台培育新型主体，集约要素建好现代园区。全年农业总产值339.7亿元，增长5%、位居全省第五。粮、油总产分别达103.2万吨、19.2万吨；生猪出栏406.9万头，茶叶、蔬菜、水果产量分别达3.6万吨、227.2万吨和45.6万吨。新发展农民专业合作社471个，新认定省级示范家庭农场32个。洋县跻身全国首批有机产品示范县行列。解决了33.26万人饮水安全问题，转移农村富余劳动力84万人。

　　（四）旅游三产快速增长。强力推进“三建设一整治”，全面实施“全域旅游工程”。18个重点旅游项目完成投资12.98亿元，市级10大文化旅游项目加快建设，勉县诸葛古镇主体竣工即将投运，留坝“水世界”建成开园。全市接待游客2915万人次，旅游总收入152.8亿元，分别增长11%和16.3%。新培育限上贸易单位76户。社会消费品零售总额实现319亿元，增长13.3%、位居全省第五。外贸进出口总额8796万美元，实际利用外资2000万美元。

　　（五）城镇建设步伐加快。坚持精品建设、产城融合、社保均等、精细管理，持续推进“城乡统筹工程”。中心城区边界划定工作进展顺利，宜居城市建设规划、地下管线综合规划等10余项规划编制全面完成。中心城区36个城建重点项目完成投资31亿元，滨江新区、兴元新区和南一环西段、燕子河整治等项目加快建设；县城“五大工程”持续推进；25个省、市级重点镇和文化旅游名镇建设亮点纷呈，完成投资31.38亿元；“美丽乡村”、新型农村社区建设取得新进展。汉中荣获“美丽中国·最美城镇”奖。

　　（六）民生事业成效显著。统筹推进“民生改善工程”、“文化振兴工程”、“扶贫攻坚工程”和“治理创新工程”，兜底补短办好民生实事。全年民生类财政支出215.3亿元，占总支出的83.3%。市政府承诺办好的“10件民生实事”全面完成。职教集团和职业教育联盟挂牌成立，公办幼儿园占比达到52.9%，138所标准化学校建设如期完成，营养计划惠及25.5万学生，高考一本上线率居全省第二。11所重点镇卫生院基本建成，人口自然增长率2.68‰。申请专利1418件，科技成果登记评审37项。养老、医疗、低保等社会保障工作扎实有效。再次荣获全省“双拥模范城”称号，“汉中舰”命名文件正式下达。精准扶贫成效明显，全年脱贫15.3万人。城镇新增就业5.8万人，城镇登记失业率控制在3.21%。公共文化、体育设施不断完善，羌文化保护中心、市体校基本建成，组建了全省首家跨多企多业的汉中文化传媒集团。校园足球和群众性文体活动广泛开展。《汉颂》、《韩信拜将》入选中国第十一届艺术节重点展演剧目。陕南移民搬迁2.8万户，公租房竣工3.94万套，农村土坯房改造7160户，汉江综合整治建成堤防主体30公里，建设通村水泥路417公里。安全生产、食品药品监管扎实有效，社会治安管理、矛盾纠纷化解取得新成效，防范处理邪教问题工作受到国家部委联合表彰，人民群众治安满意率位居全省第二。

　　（七）生态建设亮点纷呈。大力实施“生态环境工程”，铁腕治理环境突出问题，系统推进生态文明建设。循环经济加快发展，资源能源利用水平不断提升。重点减排项目建成投运42个，关停小皂素企业27家，拆改燃煤锅炉677台，秸秆禁烧、扬尘治理成效明显，中心城区空气质量二级以上天数达到282天，超额完成省上下达任务。植树造林20万亩，治理水土流失850平方公里。有力应对了陇南尾矿库泄漏引发的锑污染事故，受到环保部表扬，为省、市赢得了荣誉。

　　（八）政府建设不断加强。坚决贯彻“八项规定”精神，驰而不息反对“四风”，扎实开展“三严三实”专题教育，市级机关公务用车改革全面完成，“三公”经费同比下降28%；出台了政府性债务、产业资金、国有资产配置等管理办法，政府运行管理机制进一步健全完善。自觉接受人大、政协监督，全年邀请省、市人大代表、政协委员162人次列席市政府重要会议；人大代表建议、政协委员提案办结率100%、满意率99%。“人民网留言”和“市长信箱”来信办结率99%。市行政服务中心受理审批事项办结率95%。群众对市政府工作满意度列全省第四位。

　　审计、统计、人防、外侨、质监、民族、宗教、对台、档案、地震、气象、慈善、老龄、地方志、预备役和残疾人等工作取得新成绩。

　　各位代表！过去一年工作的全面完成，为“十二五”发展画上了圆满句号。五年来，我们主动适应经济发展新常态，实施“六大战略”优化发展路径，培育“六大产业”增强发展后劲，推进“十大工程”厚植发展基础，建好“五大园区（村）”构筑发展平台，打造“两汉三国·真美汉中”品牌提升竞争魅力，使汉中发展站在了新的历史起点上。

　　——综合实力实现整体跃升。主要经济指标位于毗邻地区前列，增速稳居全省第一方阵，生产总值年均增长12.9%，实现翻番。全面实现了生产总值、工业总产值、全社会固定资产投资、财政总收入“四个一”目标。三次产业结构进一步优化，由“十一五”末的21.7:39.1:39.2调整为18:44:38，装备制造、现代材料、绿色食药产值占规模以上工业总产值的比重分别达18.9%、35.1%和28%。中国驰名商标、地理标志商标、陕西名牌产品累计分别达到6件、12件、61个。连续两年荣获全省年度目标责任考核优秀等次。

　　——基础设施取得重大突破。全社会固定资产投资累计完成3509.9亿元，是“十一五”的3.8倍。汉中城固机场顺利通航，十天高速和宝汉高速汉中以南段建成投运，西成客专汉中段建设进度超过80%，实现了汉中环城高速的历史性突破，立体交通体系初步形成。水利、电力、天然气管网建设成效明显。中心城区一大批标志性工程竣工投用，县城、重点镇和美丽乡村建设取得显著成效，全市城镇化率达46.64%、比“十一五”末提高9.56个百分点；成功创建为国家卫生城市和省级生态园林城市、省级文明城市。

　　——生态文明建设步伐加快。启动汉江流域污染防治三年行动计划，“抑尘、减煤、治源、控车、禁燃、增绿”多管齐下，节能减排任务超额完成。中心城区生活污水、垃圾处理率分别达91.2%、100%，县城分别达89.1%、98.1%；中心城区空气质量优级天数位居全省第一；汉江、嘉陵江出境水质分别稳定保持在国家Ⅱ、Ⅲ类标准。完成造林132.5万亩，治理水土流失4468平方公里。

　　——人民生活水平明显提升。城镇居民人均可支配收入比“十一五”末增长84.4%，农民人均纯收入实现翻番。高考上线率实现“四连升”，5个县荣获国家义务教育发展基本均衡县，6个县荣获省“双高双普”县。覆盖城乡的医疗服务体系基本建成。张骞墓成功入选世界文化遗产名录，建成6个国家4A级景区。54.8万农村贫困人口实现脱贫，解决135.3万群众饮水困难。汉江综合整治建成堤防主体157.7公里。陕南移民搬迁12.23万户，建成保障性住房9.18万套，棚户区改造5.48万户，三类政策性住房覆盖全市总户数的20.4%。城镇居民人均住房面积36.5平方米，居全省前列。养老、医疗、低保、救助等社保政策实现城乡全覆盖，人民群众获得感普遍增强。

　　——改革开放激发生机活力。市级部门行政审批事项由520项精简到221项，精简率57.5%。行政、财税、文化体制改革和事业单位分类改革、镇村综合改革，以及商事制度、食品药品监管体制改革等成效显著，走在全省前列；教育体制改革受到中、省肯定，医药卫生体制改革形成了“汉中模式”。招商引资累计到位资金1800亿元，年均增长25%。中航、中建、中交建、中水建、中电建、陕煤化、陕有色等世界或国内500强企业和一大批重大基础设施类、产业类项目相继落户汉中，为全市发展注入了强劲持久动力。

　　各位代表！过去五年，我们深化市情认识、顺应发展大势，在艰辛探索中开辟了“三市”建设新路径；我们奋力抢抓机遇、沉着应对挑战，在顽强拼搏中开创了追赶超越新局面；我们坚持改革创新、激活发展动力，在突破瓶颈中开拓了转型升级新空间；我们担当为民使命、全力改善民生，在共建共享中开启了全面小康新征程。五年历程充满艰辛，五年成就令人鼓舞。这是在省委、省政府和市委的坚强领导下，市级各大班子凝神聚力、全市各级党政组织开拓创新的结果；是全市广大干部群众苦干实干的结果。在此，我代表市人民政府，向奋战在各条战线的工人、农民、知识分子、干部、驻汉部队指战员、武警官兵和公安干警致以崇高的敬意！向大力支持政府工作的各位代表、各位委员和同志们、朋友们表示衷心的感谢！

　　在充分肯定成绩的同时，我们也清醒地认识到，汉中未来发展还存在一些矛盾、困难和短板：宏观经济下行，环境约束增强，建设用地趋紧，企业融资困难，追赶超越面临很大压力；传统产业占比偏高，新兴产业支撑有限，科技创新能力不足，转型升级仍然任重道远；城乡居民收入不高，贫困人口量多面广，公共服务水平较低，全面建成小康任务艰巨；少数干部作风不实、为政不廉、不敢担当和为官不为、为官慢为等问题依然存在。对此，我们必须高度重视，主动作为，认真加以解决。

　　二、“十三五”发展的基本思路和目标任务

　　“十三五”时期，是全面建成小康社会的决胜阶段，也是实现“三市”目标的关键时期。我们要把适应新常态、把握新常态、引领新常态作为贯穿发展全局和全过程的大逻辑，认真学习贯彻党的十八大以来中央各次全会和习近平总书记系列讲话精神，全面落实“五位一体”总体布局和“四个全面”战略布局，牢固树立“五大发展理念”，崇尚创新、注重协调、倡导绿色、厚植开放、推进共享，坚持追赶超越、绿色循环、转型升级工作主基调，深入实施“六大战略”、发展壮大“六大产业”，持续推进“十大工程”、建设“五大园区（村）”，坚决打赢脱贫攻坚战，做优叫响“两汉三国·真美汉中”城市品牌，在变中求新、新中求进、进中突破，推动汉中发展不断迈上新台阶。总体目标是：在提高发展质量效益的前提下，实现经济中高速增长，GDP年均增速10%以上，高于全省平均水平2个百分点；全社会固定资产投资年均增长20%；规模以上工业增加值年均增长12%以上；社会消费品零售总额年均增长10%以上；地方财政收入同口径年均增长10%以上。到2020年，生产总值超过1750亿元、人均力争达到8000美元，全体居民收入增速高于全省平均水平，基本公共服务实现均等化，“三市”建设迈上更高水平，汉中与全国全省一道同步够格全面建成小康社会。

　　实现上述奋斗目标，我们必须崇尚创新，着力推进发展动力转换，力促发展方式从粗放增长转向集约增长，让新技术、新产业、新业态蓬勃发展；必须注重协调，着力增强发展整体效能，促进区域协同、城乡一体、“四化”同步和经济社会发展协调、物质精神文明并重；必须倡导绿色，着力推进人与自然和谐共生，加快构建科学合理的城乡一体化格局、现代农业发展格局、生态安全格局、自然岸线格局，走生产发展、生活富裕、生态良好的文明发展之路；必须厚植开放，着力打造丝绸之路源点城市，加快培育开放型现代产业体系，打造开放开发新高地；必须推进共享，着力增进人民群众福祉，加快补齐小康短板，让全市人民在共建共享发展中有更多获得感。

　　根据上述要求，“十三五”期间，我们要重点在六个方面实现新跨越：

　　（一）经济转型升级跃上新台阶。坚定走创新驱动、转型升级、循环发展之路，通过技术引进、消化吸收和再创新，集中力量突破一批支撑引领发展的关键技术；主动顺应“中国制造2025”、“互联网+”等转型升级新要求，改造提升传统产业，大力发展特色优势产业，促进三次产业融合发展，做特现代农业、做强新型工业、做优现代服务业；大力发展绿色循环经济，打造12条循环经济产业链和6大循环农业产业链，全力构建“一心两带三板块六集群七极支撑”的循环产业新格局。到2020年，三次产业结构调整到12:49:39；规模以上工业总产值突破2000亿元大关，农业总产值达到695亿元，旅游总收入突破300亿元；战略性新兴产业增加值占生产总值比重超过15%，非公有制经济比重保持在52%以上。

　　（二）基础设施建设实现新突破。拓展汉中城固机场航线布局，建成西成客专、阳安二线、宝汉高速等项目，推进汉巴渝及北上通道快速铁路、西乡至镇巴高速公路、中心城区城市轻轨等项目建设，实现县县通高速、重点镇和4A级景区通等级路、具备条件的行政村通沥青（水泥）路。大力实施汉江综合整治、引汉济渭等工程，云河水库建成投运；加快引嘉济汉工程、城固焦岩水库和略阳双庙崖水库前期工作。持续抓好电源电网、新能源建设和清洁能源推广，建成汉中电网330千伏第二电源通道，“气化汉中”基本实现市、县、重点镇全覆盖。统筹推进新一代信息基础设施、防灾减灾基础设施建设。

　　（三）城乡统筹发展再创新业绩。按照“一个尊重、五个统筹”要求，坚持“盆地聚集、中心做大、两翼伸展、沿江发展”城市建设思路，依托“五大形象设计”全面塑造城市特色，加快中心城区“一江两岸”、滨江新区、兴元新区、龙岗片区建设，不断提升综合承载能力和宜居性；引导县城建设完善功能、改善环境、凸显特色、提升品位，推动南郑、城固撤县设区和勉县、西乡县撤县设市；提升重点镇的产业配套水平和人口集聚功能，建好500个美丽乡村。实施“智慧汉中”建设，推进城市管理规范化、精细化、网络化、智能化，成功创建国家园林城市。2020年全市常住人口城镇化率达到58%以上。

　　（四）生态文明建设取得新进展。以建设环境保护、产业发展和新型城镇化同步推进的国家生态文明综合改革示范区为载体，抓好大气、水、土壤污染防治，加快市、县污水处理厂提标改造，持续开展农村环境综合整治和面源污染治理，强化水源地保护，确保汉江、嘉陵江水质稳定达标。着力构建现代农业、新型工业、现代服务业和社会循环四大循环体系，节能减排完成省上下达任务。抓好汉江流域生态修复与保护工程、丹江口库区上游水土保持治理工程，森林覆盖率达到60%以上，建成国家森林城市、环保模范城市和生态文明建设示范市。

　　（五）改革开放事业再谱新篇章。全面推进经济体制、要素市场、行业管理和重点领域改革。顺应经济全球化潮流，推进全方位开放，更好融入“一带一路”、长江经济带和京津冀经济圈，打响“中国汉中·张骞文化经贸使团”品牌，引进更多的世界500强、国内500强、行业100强企业落户汉中。争取我国西行国际列车、西成客专相关车次被命名为“张骞号”、“熊猫号”，不断扩大汉中对外影响力。大力发展外向型经济，探索建立电子口岸平台，促进外贸进出口龙头企业快速成长。到“十三五”末，全市外贸进出口总额突破2亿美元，吸引外商直接投资2亿美元；招商引资到位资金年均增长12%左右。

　　（六）城乡人民群众共享新成果。践行以人民为中心的发展理念，坚持不懈保障和改善民生。新增就业17万人。普及学前三年教育，全面实施13年免费教育，推进中等职业教育逐步免费。社会保障实现基本制度定型完备、法定人群全面覆盖、保障标准合理调升目标，建立确保困难群众基本生活的社会救助体系。实施“健康汉中行动计划”，深化公立医院综合改革，2017年县域就诊率达到90%左右。完善全民健身服务体系，加快公共文化设施建设。扎实推进陕南移民搬迁、保障性住房建设和城中村、棚户区改造，不断改善群众居住环境和条件。2018年底前现行标准下的贫困人口全部脱贫、贫困县全部脱贫摘帽；到2020年，全面打赢脱贫攻坚战。

　　放眼“十三五”，前景美好，充满希望。我们一定要加倍努力，推动汉中早日实现全面建成小康社会的宏伟目标！

　　三、2016年主要工作任务

　　今年是实施“十三五”规划、开启全面建成小康社会决胜阶段的起始之年，也是推进供给侧结构性改革的攻坚之年。综合分析当前形势，我们既要看到前进道路上的许多困难和问题，更要看到面临的大好机遇和有利条件：党的十八届五中全会、中央经济工作会提出的发展新理念和一系列战略举措，为我们开启“十三五”发展引领了航向、提供了强大支撑；“一带一路”、脱贫攻坚等国家重大战略的全面深入推进，为我们提供了十分有利的政策机遇；经过“十二五”的快速发展，我市经济总量持续扩张，综合实力大幅提升，基础设施显著改善，立体交通网络、骨干产业体系基本形成，尤其是航空智慧新城、滨江新区、兴元新区等一大批重大项目加快推进，汉中发展潜能将加速释放。我们要乘势而上、奋发有为，牢牢掌握工作主动权。

　　今年全市经济社会发展的预期目标是：生产总值增长10%左右，全社会固定资产投资增长20%以上，规模以上工业增加值增长11%左右，地方财政收入同口径增长10%以上，社会消费品零售总额增长11%，城镇和农村常住居民人均可支配收入分别增长11%、12%左右，居民消费价格指数控制在3%以内，人口自然增长率控制在6‰以内，单位GDP能耗下降3.6%。

　　围绕上述目标，重点抓好八个方面的工作：

　　（一）紧扣中高速增长，实现经济健康发展。

　　强化投资拉动作用。大力推进“千亿投资工程”，全年实施重点项目332个，完成投资428.2亿元，建成汉安与中贵输气管道联络线、国网110千伏及以下电网建设等25个项目，加快建设西成客专、阳安二线、宝汉高速、引汉济渭、酒泉至湖南特高压线路汉中段工程等95个在建项目，新开工西乡至镇巴高速公路、兰海高速青木川连接线等86个项目，积极推进汉巴渝铁路等126个前期项目，带动全社会固定资产投资完成1247亿元。

　　力促工业较快发展。加快实施新一轮“工业翻番工程”，全面落实工业稳增长的各项政策，促进市内钢铁、有色、建材产品与基础设施建设采用对接，支持企业建立电商销售平台，不断拓宽销售渠道。推进骨干企业和小微企业协作配套，引导企业推行精益管理，千方百计提高生产经营效益。实施50个工业技改项目，做好新增产能项目的跟踪服务，促进秦元新材料、天然谷保健品生产线等项目尽早投产达效。加快航空智慧新城、桑溪工业园、黄玉产业园建设，提升工业集群化发展水平。

　　培育新的消费热点。围绕消费新需求，增加优质新型产品和生活服务等有效供给，培育壮大健康养生、医疗康复、养老休闲、文化娱乐等消费业态。积极探索新型营销模式，全面加快电子商务进农村步伐，促进电商、网购、快递等融合发展。继续提高住房公积金使用率，优化工作流程，支持住房消费。严格落实职工带薪休假制度。继续开展“汉中旅游惠民活动”，激活本地群众消费潜能。全年社会消费品零售总额完成353亿元。

　　（二）突出创新驱动，加快产业转型升级。以创建国家创新型试点城市为契机，以质量和品牌强市战略为引领，推进产学研战略联盟、示范基地、示范项目建设，搭建设施平台、技术平台和信息平台，大力发展新技术、新产业、新业态，促进产业向中高端发展。

　　支柱工业高端化。以航空产业为主体，加大航空智慧新城军民融合力度，推动产业项目建设，扩大聚集规模。支持机床工具、输变电设备、汽车及零部件、机械设备、智能制造等横向联合，推动装备制造业向智能化、系统化发展。规范矿产资源开发，提高利用水平，优化产品结构，整合延长钢铁和有色产业链，大力发展新型现代材料产业。依托汉中绿色资源，培育具有较强竞争力的有机食品、功能食品、植物提取、中医药等产业，打造国内知名的绿色有机食药品牌基地。加快镇巴石油天然气勘探步伐，积极争取国家投资开发。

　　全域旅游显特色。围绕建设国际自然旅游目的地和彰显“两汉三国·真美汉中”主题，加快实施“全域旅游工程”规划，加大华阳、紫柏山、黎坪景区创5A和五龙洞、石门栈道、午子山等景区创4A步伐，推进华阳、黎坪景区快速专用道路和“一江两岸”5A级景区、大汉山、红寺湖风景区全面建设，持续抓好“三建设一整治”，优化旅游线路，大力提升休闲农业和乡村旅游，加强旅游商品开发。全年接待游客3200万人次、增长10%以上，实现总收入172亿元、增长12%以上。

　　三产服务上水平。推动生产性服务业向专业化和价值链高端延伸，生活性服务业向精细化和高品质转变，使服务业成为发展新引擎。大力发展信息服务、研发设计、融资租赁等新兴服务业，改造提升商贸、住宿、餐饮、交通运输等传统服务业。加快褒河物流园区及各县区物流节点的建设步伐，推进亿丰国际商贸城等城市商业综合体、特色商业街区建设。搭建政、银、企合作平台，引导商业银行扩大信贷投放。抓好县域金融综合改革试点，加快发展民营银行。

　　新兴产业求突破。积极落实扶持战略性新兴产业发展的财税金融政策，促进大数据、云计算、物联网、3D打印等新技术在各个领域的推广应用，推动新一代信息技术与各行业的深度耦合，不断探索融合发展的新业态、新模式，打造战略性新兴产业集群，加快培育新的经济增长点。

　　（三）致力提质增效，大力发展现代农业。加快农业供给侧结构性改革，下功夫调整种养结构，扩大优质农产品生产，提升农业经济效益，使“农业倍增工程”向纵深推进。

　　推动集约发展。实施藏粮于地、藏粮于技战略，稳定种植面积，粮、油总产分别保持在100万吨、17万吨以上。加快土地流转，引入社会资本，发展规模经营。以特色化、高端化为方向，全力打造全省十大绿色农产品生产基地，推进特色产业不断扩大规模、提高效益。全年生猪出栏410万头，茶叶、蔬菜产量分别超过3.7万吨、220万吨。

　　提高综合效益。强化科技支撑、资本运作、人才引进，支持市场前景好、带动作用强的粮油、畜禽、茶叶、果菜、药菌等加工企业做优品质、做强品牌，提升效益和竞争力。大力培育家庭农场、职业农民等新型经营主体，提升现代农业园区经营管理水平。扶持营销主体，培育专业市场，带动农民分享产业增值效益。加强与京津冀地区、“一带一路”沿线城市的交流合作，推动汉中农产品走向更广阔市场。

　　增强发展后劲。加大涉农资金的整合力度，提高资金使用效益，发挥财政资金对结构性改革的引导作用，撬动更多社会资金投入农业农村。加强农村支付体系和信用体系建设，积极探索金融支农惠农新模式，有效缓解农村融资难、贷款难、贷款贵等问题。深化农村产权制度改革，完成农村承包土地确权登记颁证工作，搞好农村承包土地经营权抵押贷款试点。抓好第三次农业普查工作，坐实农业农村各项基础性数据。持续开展农田水利、土地整治、中低产田改造和高标准农田建设，加快实施“坡改平”、“旱改水”工程，夯实农业发展基础。

　　（四）坚持城乡统筹，强力推进新型城镇化。认真贯彻落实中、省城市工作会议精神，持续实施“城乡统筹工程”，不断提升以人为核心的新型城镇化水平。

　　更新理念，科学谋划。不断学习和创新规划理念、方法，综合考虑城市功能定位、文化特色、建设管理等多种因素，大力推进城市设计，加强城市空间立体性、平面协调性、风貌整体性、文脉延续性等方面的规划和管控，强化汉中特有的地域环境、历史文化、建筑风格等特色“基因”传承，推广应用海绵城市和地下综合管廊建设技术，持续完善路网、绿地、公厕、停车场、水电气等基础设施，提升城镇宜居水平和辐射带动能力。

　　重点突破，打造精品。今年中心城区实施城市基础设施重点项目82个，完成投资301.54亿元。高标准抓好滨江新区、兴元新区、龙岗片区等区域开发，加快实施火车站南广场改扩建、天汉文化公园等重点项目。抢抓棚户区改造历史机遇，用足用好政策资金，加快老城区改造，大幅提升市民居住条件和城市服务水平。大力推进县城扩容提质，高水平建设公共服务设施和地下管网，提高集聚要素和带动镇村能力。加快提升25个重点镇、文化旅游名镇，培育一批交通节点型、旅游度假型、资源加工型、商贸流通型特色城镇。继续抓好100个美丽乡村示范村、20个旅游精品村，新建35个新型农村社区，新启动300个美丽乡村和10条美丽乡村风景线（带）建设，努力打造村容整洁、文化浓郁、生态优美、产业支撑、设施配套的新农村。

　　综合施策，精细管理。以实施居住证制度为抓手，推动城镇常住人口基本公共服务均等化；以“四城联创”为载体，美化市容环境，综合整治群众关注的交通拥堵、物业管理不规范、乱挖乱建和乱停乱放等突出问题。建立健全城镇水、电、气供应应急机制，有效保障群众生活。着手开展智慧城市建设，抓好数字化平台建设和功能整合，使城市管理更精细、更便捷、更高效。

　　（五）聚力生态宜居，不断提升环境质量。以“汉江流域污染防治三年行动”决战决胜为目标，大力实施“生态环境工程”，力争建成省级环保模范城市。

　　铁腕治污降霾。抓好已建污水处理厂提标改造，加快沿汉江重点镇污水处理设施建设，开工建设铺镇污水处理厂和垃圾焚烧发电厂，下功夫治理汉江及其支流南郑濂水河、城固堰沟河、宁强小河污染以及略阳麻柳铺坑涌废水；大力开展城乡垃圾清理、重点行业除尘工程升级改造、燃煤锅炉拆改、建筑施工和道路扬尘污染防控，加大“黄标车”、老旧车淘汰力度，实施挥发性有机物整治，保证汉江、嘉陵江出境断面水质持久达标，中心城区空气质量达到省考指标。

　　严格节能减排。支持钢铁、水泥、电力、化肥等重点行业实施节能技术改造，推行风能、太阳能、生物质能、水能等清洁能源的开发利用，全年消纳清洁能源9.5亿千瓦时。出台支持电能替代等政策措施，实现电能替代2.26亿千瓦时，高标准建设一批电动汽车充电站。继续实施重点行业、重点企业“三废”处理设施提标改造，努力削减排污总量。全年化学需氧量、氨氮分别削减1%，二氧化硫、氮氧化物分别削减2%。

　　强化生态建设。统筹实施土地“双退”、天然林保护等生态工程，大力推进汉江综合整治、水土流失治理和汉江支流入口湿地群建设，完成造林22万亩，汉江综合整治新修、加固堤防30公里，治理水土流失850平方公里。

　　开展绿色创建。在城乡社会广泛开展绿色庭院、绿色社区、绿色企业、绿色校园等群众性绿色创建活动，鼓励绿色居住、绿色出行、绿色旅游，让绿色成为全社会的共识和行动。

　　（六）精准发力施策，打好脱贫攻坚战。全面实施“脱贫攻坚工程”，确保全市脱贫15.5万人、佛坪县率先脱贫摘帽。

　　完善脱贫规划。贯彻中、省精准扶贫、精准脱贫的决策部署，出台我市脱贫攻坚《规划》。及时跟进中省《川陕革命老区振兴发展规划》编制工作，加大汇报衔接力度，争取我市有更多项目进入中省盘子、早日开工建设。

　　加大资金投入。大力实施《1+19配套支持计划》，形成齐抓共管格局；全面推进金融扶贫，全年投放金融扶贫贷款超过10亿元，建立扶贫互助资金协会100个；以整村推进、扶贫移民搬迁为抓手，实施一批基础设施和民生项目，进一步改善贫困村生产生活条件。全年建设高标准农田6万亩，建成产业化项目30个。

　　统筹协调推动。按照“五个一批”的精准脱贫要求，统筹采取产业和就业扶贫、劳务输出、移民搬迁、生态补偿、教育扶贫、健康扶贫、兜底保障等综合措施，精准滴灌、靶向施策。突出“六个精准”、“双联双包”，完善市、县、镇三级驻村帮扶制度，充分发挥干部包扶、驻村工作队包抓和“第一书记”直接责任人的作用，鼓励社会各界广泛参与，使各项脱贫措施到户到人、到田间地头。

　　（七）立足共建共享，全力增进群众福祉。持续实施“民生改善工程”、“文化振兴工程”、“治理创新工程”，更好发挥社会保障的“稳定器”作用，让人民群众有更多获得感。

　　不断健全社保体系。大力开展大众创业、万众创新，完善创业孵化基地和就业优惠政策，重点做好高校毕业生、移民搬迁户、“零就业”家庭等特殊群体就业扶持工作，全年城镇新增就业3.2万人，农村富余劳动力转移就业75万人次，城镇登记失业率控制在3.9%以内。持续扩大社会保险覆盖范围，织牢社会保障安全网底。落实困难残疾人生活补贴和重度残疾人护理补贴制度，抓好城乡低保、农村五保和社会救助、医疗救助。应对老龄化趋势，加快发展养老事业。

　　全面发展社会事业。认真贯彻全省教育工作会议精神，大力实施第二轮教育“五大工程”，持续抓好营养改善、优质教育资源扩面、“双高双普”县创建和整合职业教育等工作，支持汉中职业技术学院创建省级示范高职学院。深化市、县公立医院改革，加强重点传染病防控、妇幼和中医专科、镇卫生院能力建设。全面实施一对夫妇可生育两个孩子政策，切实落实失独家庭补助救助政策。继续推进公共体育设施建设，广泛开展全民健身活动。

　　大力推进文化建设。挖掘利用“两汉三国”文化、秦巴民俗文化、红色革命文化等地域文化，支持文艺创作和社会组织发展文化事业，讲好“汉中故事”，展示文化魅力。不断完善公共文化服务体系，抓好重点镇综合文体中心、村文化室等项目，广泛开展丰富多彩的城乡群众性文化活动，积极推进工人文化宫、青少年宫、妇女儿童中心项目建设。精心打磨提升《汉颂》、《韩信拜将》等精品剧目，力争在中国第十一届艺术节上获奖。加快国家级羌族文化生态保护实验区建设，做大做强汉中文化传媒集团，大力推动扶持各类文化产业与其他产业融合发展，全年文化产业增加值增长10%以上。

　　切实维护和谐稳定。大力开展安全生产精细化管理、标准化建设、新技术推广，坚决杜绝重特大事故，有效遏制较大事故，努力减少一般事故。构建和谐劳动关系，维护劳动者合法权益。综合运用法律、行政、经济和教育引导的方法，规范信访秩序，预防和化解社会矛盾。落实“四个最严”要求，继续完善市、县、镇三级食药监管责任体系，确保食品药品安全。全面开展“七五”普法，创新完善立体化社会治安防控体系，严厉打击各类违法犯罪活动，建设“平安汉中”。

　　（八）深化改革开放，促进发展行稳致远。坚持全面深化改革开放不动摇，为经济社会发展添动力、增活力、释放红利。

　　加快供给侧结构性改革。正确处理供给与需求、打好歼灭战与持久战、市场主导与政府引导、推进改革与社会稳定的关系，在推进供给侧改革上求突破。在去产能方面，分类推进国企改革，优化国有资本配置，积极发展混合所有制经济；争取中省专项奖补资金，加快淘汰“僵尸企业”、高污染企业和产能过剩领域无竞争力企业，妥善安置分流人员；下决心削减钢铁的同质化产能，整合产业链，调整下游产品结构，发展钢构产业；淘汰粮油粗加工、粘土实心砖、小石灰、小化工等落后产能，全力打好“去产能”歼灭战。在去库存方面，分类施策支持有市场有效益企业扩大销售，推动企业开发适销对路产品，积极扶持小微企业健康发展。特别要打通并融合保障房、商品房、移民搬迁房政策，加大库存消化力度。在去杠杆方面，做好地方政府存量债务置换工作，完善全口径政府债务管理；加强全方位监管，规范各类融资行为，打击非法集资，维护群众合法权益。在降成本方面，从降低交易、人工、物流、财务成本等方面发力，打好降本增效组合拳。在补短板方面，切实解决好贫困以及“三农”、生态、基础设施等方面存在的问题，以全要素生产率提高促进经济增长。同时，继续深化行政审批、财税体制、预算制度、商事制度改革，完成镇村综合改革任务。

　　围绕市场化目标配置要素。改革科技成果使用、处置、收益管理办法，积极培育综合性专利运营企业，探索科技贷款风险补偿和知识产权质押融资模式，充分释放创新要素活力。创新财政投入方式，通过设立产业发展基金、推行股权投资、搞好以奖代补、开展风险补偿、完善财政贴息和政府购买服务等方式，吸引更多社会资本投向优势产业。支持企业上市融资或发行债券，促进金融更好服务实体经济。继续加大闲置土地处置力度，盘活存量，提高效益。

　　全方位扩大对外开放。依托装备制造、矿产开发、生态旅游、绿色食药、“两汉三国”文化等优势，统筹推进与丝路沿线国家的交流合作，推动产能和产品“走出去”、资金和游客“走进来”。组织企业开展外贸投资洽谈活动，加快汉中口岸、海关建设，扩大外贸总额。深化与北京、上海等有关方面战略合作，努力在承接产业转移和特色产品走出去上取得新突破。瞄准世界500强和京津冀、“长三角”、“珠三角”产业转移新动向，大力开展产业链招商和叩门招商、以商招商，强化招商引资的针对性和项目跟踪服务工作。全年引资到位500亿元。

　　各位代表，今年市政府承诺继续为全市人民办好10件实事：一是建设200个农村互助幸福院，20个社区养老服务站和城市社区日间照料中心，扶持10个民办养老机构；二是建成200所标准化学校，创建2个“双高双普”县区、2个国家义务教育基本均衡县；三是改造土坯房1.8万户；四是实施棚户区改造1.4万户；五是移民搬迁2万户；六是新建、改建农村公路450公里；七是实施350个村级农民体育健身工程；八是为2.5万对符合两孩生育政策、有生育意愿的夫妇提供免费孕前优生健康检查；九是为群众购买文化惠民演出600场、公益数字电影3万场次；十是中心城区新建或改造生活垃圾收集站20个、小型压缩站2个、公厕54个、集贸市场13个。

　　四、加强政府自身建设

　　历史的使命、发展的重任和人民的期待，要求我们必须自觉践行“三严三实”，深入开展“两学一做”活动，紧扣“三讲三当”主题，深化作风建设，不断提升贯彻新理念、引领新发展的能力和水平，坚决做到面对困难要有信心、面对调整要有对策、面对挑战要有能力、面对任务要有担当，以忠诚、担当、务实、干净的形象取信于民，最大限度地为发展集聚正能量，让发展释放正效应。

　　聚焦忠诚，建设行为规范的法治政府。严守党的政治纪律和政治规矩，认真贯彻中省和市委决策部署，做到表里如一、知行合一。强化民本意识，心系群众冷暖，做好事、办实事、解难事，努力使全市人民在共建共享中有更多幸福感。严格按照法定权限、法定程序行使职权、履行职责，自觉接受人大依法监督、政协民主监督和社会公众监督；加快制定法治政府建设实施规划，推进政府系统机构、职能、权限、程序、责任法定化，促进严格执法、文明执法、公正执法相统一。

　　聚焦担当，建设干事创业的责任政府。牢记使命责任，勇于担当作为，大力倡导说了就办、定了就干、干就干好的担当精神，知责有为、敬业奉献。强化政风政纪督查，对责任心缺失、不作为、乱作为的政府部门及工作人员严格执纪问责。建立合理容错机制，做到“三个区别开来”，让那些作风正派、锐意进取的干部更好地带领群众干事创业。

　　聚焦务实，建设勤勉高效的服务政府。坚持低调务实不张扬，埋头苦干抓落实，聚精会神促发展。抓好政务服务中心、“市长信箱”、“市长热线”等为民服务平台建设，落实县、镇、村三级群众办事干部代办制度，不断提高政府服务效能。全面公布市县两级部门权力清单和责任清单，落实好负面清单制度。继续减少和规范行政许可事项，实行投资项目审批监管线上运行。推进监督执法重心下移，落实属地监管职责，切实解决市场监管、安全生产、食品药品安全、市容市貌、社会诚信的管理缺位、力量不足等问题。

　　聚焦干净，建设风清气正的廉洁政府。自觉履行“两个责任”，严守廉洁自律准则和党纪处分条例，始终把纪律挺在前面。持之以恒落实“八项规定”精神，健全厉行节约反对浪费制度体系，着力解决群众身边的不正之风，持续发力转作风、促政风、带民风。完善惩治和预防腐败体系，规范工程招投标、施工预算、土地矿产资源利用、政府采购、国有产权交易等公共资源管理，切实加强审计监督，严厉查处权力寻租、权钱交易和损害群众利益的行为，树立为民、务实、清廉的政府形象。

　　各位代表，“十三五”的蓝图已经绘就，全面小康的号角已经吹响。让我们在市委的坚强领导下，同心同德，锐意进取，求真务实，埋头苦干，为实现全面建成小康社会宏伟目标而努力奋斗！
